# Diagnostic Pitfall of Platypnea-Orthodeoxia Syndrome Caused by Atrial Septal Defect after Right Pneumonectomy

**DOI:** 10.1155/2020/4257185

**Published:** 2020-02-20

**Authors:** Y. Bohren, M. Lopez, N. Santelmo, J. Ristorto, P. Billaud, M. Canuet, P. M. Mertes, O. Collange

**Affiliations:** ^1^Department of Anaesthesiology and Intensive Care, Hôpitaux Universitaires de Strasbourg, Strasbourg, France; ^2^Department of Thoracic Surgery, Hôpitaux Universitaires de Strasbourg, Strasbourg, France; ^3^Department of Cardiology, Hôpitaux Universitaires de Strasbourg, Strasbourg, France; ^4^Department of Cardiac Surgery, Hôpitaux Universitaires de Strasbourg, Strasbourg, France; ^5^Department of Pulmonology, Hôpitaux Universitaires de Strasbourg, Strasbourg, France

## Abstract

We describe a case of platypnea-orthodeoxia syndrome (POS) due to atrial septal defect (ASD) occurring in the early postoperative course of a right pneumonectomy. Deformation of the atrial septum after right pneumonectomy deviates the blood from the inferior vena cava to ASD during the sitting position creating, a massive right-to-left shunt. Diagnosis can initially be missed by making contrast bubble test through the superior vena cava. The atrial septal defect was then closed using the surgical technique, allowing an instantaneous improvement of hematosis.

## 1. Introduction

Platypnea-orthodeoxia syndrome (POS) is a rare consequence of anatomical changes after a right pneumonectomy with the apparition of the intracardiac shunt. The patent foramen ovale (POF) is among the causes of shunting the most described [1]. We present here a case of an atrial septal defect (ASD) reopening after right pneumonectomy.

## 2. Case Presentation

A 76-year-old woman underwent a right pneumonectomy for a T3N2M0 epidermoid adenocarcinoma localized in the right upper and middle lobes. Her past medical history included high blood pressure and recurrent venous thromboembolism. According to the French legislation, the patient's nonopposition to the use of her data was obtained.

Management consisted of neoadjuvant chemotherapy followed by surgery. The preoperative evaluation revealed no contraindications, and in particular, any apparent atrial septal defect on echocardiogram was found. Since the intermediary artery was infiltrated without any possibility of sleeve artery resection, the best option for the patient was right pneumonectomy. There was no anesthetic or surgical incident during the intervention. The patient was discharged for the regular ward 5 hours after with 95% oxygen saturation on room air.

Two days later, the patient presented an acute respiratory failure associated with a severe hypoxemia. She had bronchial congestion, with polypnea and a sinusal tachycardia, but no fever. The computed tomography (CT) showed a pulmonary edema, without pulmonary embolism or infection's sign. Transthoracic echography found normal left ventricular pressures, and the improvement was fast with diuretic therapy, in favor of postpneumonectomy noncardiac edema.

After seven postoperative days, the patient presented another episode of hypoxemia, and this time increased by the sitting position with an oxygen saturation dropping from 93% in the supine position to 70% in the upright position. The blood gas showed a severe isolated hypoxemia (PaO_2_ 50 mmHg under oxygen 15 L/min). Another CT showed a pulmonary embolism. There were no clinical or radiological sign in favor of an acute heart failure or a pulmonary infection. It evoked a platypnea-orthodeoxia syndrome related to an intracardiac right-to-left shunt. Transthoracic echocardiogram with agitated saline injected in the superior vena cava (SVC) highlighted grade 1 intracardiac right-to-left shunt which cannot be responsible for the severity of hypoxemia. No ASD or POF was found.

**Figure 1:**
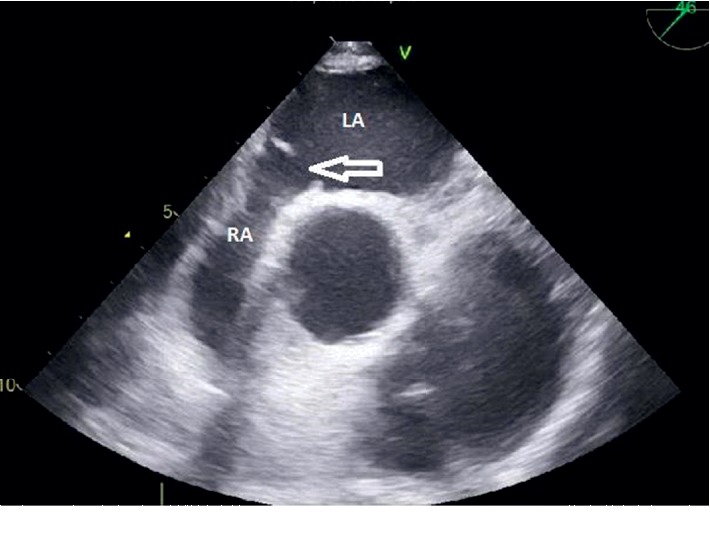
Transesophageal echocardiogram: midesophageal aortic valve short axis view of atrial septal defect. RA: right atrium, LA: left atrium.

**Figure 2:**
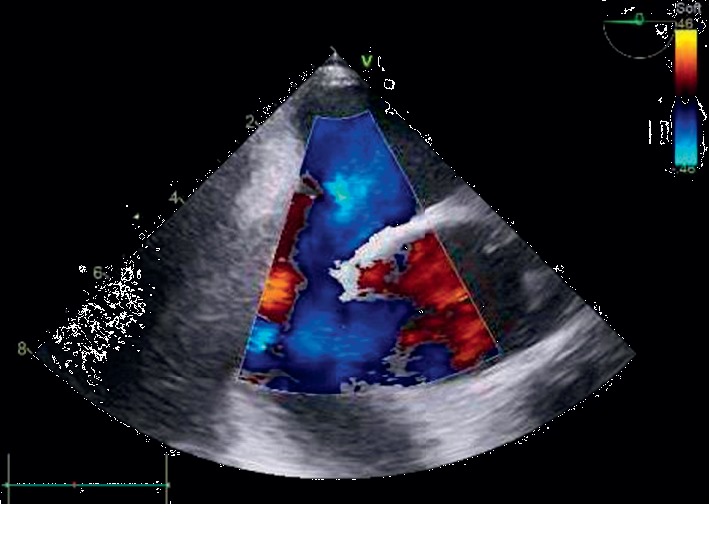
Transesophageal echocardiogram: midesophageal aortic valve short axis view of left-right shunt and atrial septal defect in the supine position.

**Figure 3:**
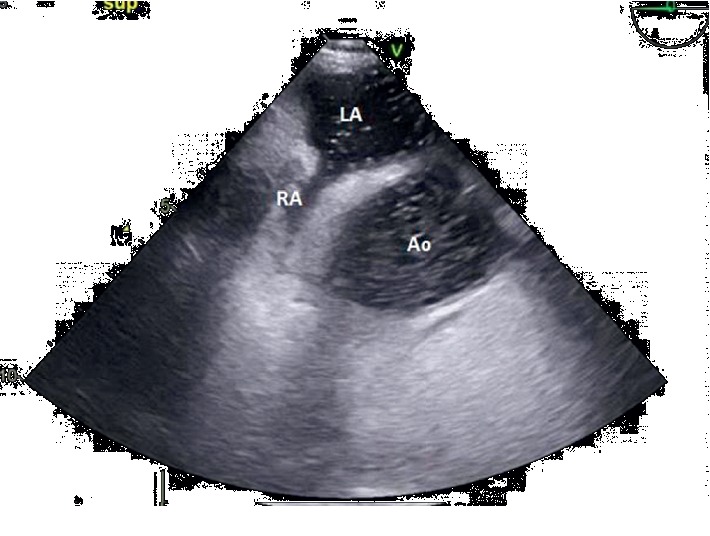
Transesophageal contrast echocardiogram: midesophageal aortic valve short axis view of atrial septal defect; superior vena cava injection in the upright position with a small right-left shunt. RA: right atrium, LA: left atrium, Ao: aorta.

**Figure 4:**
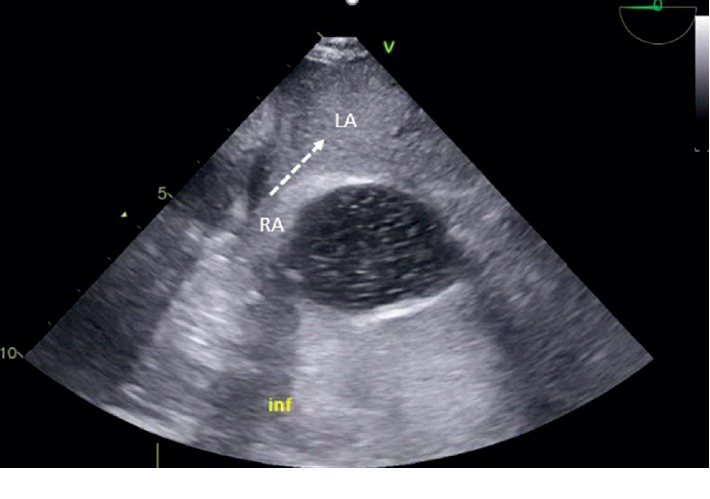
Transesophageal contrast echocardiogram: midesophageal aortic valve short axis view of atrial septal defect; inferior vena cava injection in the upright position with a massive right-left shunt. RA: right atrium, LA: left atrium.

Subsequent transesophageal echocardiogram revealed an atrial septal defect antero-inferior of 8 mm (Figure 1) with a left-right shunt in the supine position (Figure 2). Agitated saline injected in SVC during upright position highlighted grade 1 intracardiac right-to-left shunt which was insufficient to explain symptomatology (Figure 3). Therefore, the bubble test was performed in the lower cave territory through the femoral catheter during the upright position, unmasking a massive right-to-left shunt (Figure 4). No compression of the right ventricule was detected. A right catheterization in the supine position confirmed normal pulmonary pressure: systolic pulmonary arterial pressure (PAP) was 24 mmHg, diastolic PAP 9 mmHg, and mean PAP 15 mmHg under O_2_ 1.5 L/min. The right atrial pressure was 3 mmHg. The cardiac index was at 3 L/min/m^2^. Pulmonary vascular resistances were normal.

The absence of the lower edge (1 mm at the retroaortic level) makes percutaneous closure difficult, and the ASD was then closed using a pericardial patch under cardiopulmonary bypass. Her oxygen saturation on air immediately increased to 95% in the supine position and 96% in the erect position with symptomatic relief.

## 3. Discussion

The platypnea-orthodeoxia syndrome is a rare clinical entity, which must be evoked, in the differential diagnosis of a positional dyspnea and refractory hypoxemia. The most frequent causes are intracardiac shunts such as a patent oval foramen, an incomplete atrial septum, a status after lung resection [2]. ASD usually causes a left-to-right shunt. On the contrary, a right-to-left shunt is usually associated with a spontaneous or acquired pulmonary hypertension. However, in the absence of pulmonary hypertension, other mechanisms can take place.

Anatomical modifications also occur after a pneumonectomy. Indeed, in the cavity, fluid takes the place of the air 3 weeks to 7 months after. After right pneumonectomy, the diaphragmatic cupola rises several centimeters, and mediastinum performs counterclockwise rotation. The septal repositioning favors blood flow through the defect. Upright position exacerbates this stretching, and the interatrial septum is then oriented directly in the axis of the inferior vena cava (IVC) resulting in the deviation of blood flow from IVC to the atrial septal defect direction, thus leading to a massive right-to-left shunt [3].

Dynamic transesophageal echocardiogram confirms diagnosis highlighting right-to-left shunt in the upright position with color doppler and contrast test. Due to the anatomical modifications previously described, the shunt comes mainly from IVC, and the diagnosis can be missed by making bubble test exclusively through the SVC (Supplementary Materials (available ([Supplementary-material supplementary-material-1]))). To our knowledge, this pitfall has never been described in the literature.

Closure of an ASD is the best treatment of the POS [4]. When compared to surgery, percutaneous closure provides quicker recovery [5]. However, several anatomical, especially deficiency of surrounding rims, may limit its feasibility [6].

Postpneumonectomy acute respiratory failure remains a frequent and severe complication after pneumonectomy. Platypnea-orthodeoxia syndrome is a rare postsurgery complication to evoke, after classic etiologies are eliminated. The shunt comes mainly from the flow of the IVC, and the diagnosis can be missed by making contrast bubble test exclusively through the SVC in the upright position. Once the diagnosis of POS is suspected, transesophageal echocardiogram in the supine position must be performed with agitated fluid solution injected through the superior and inferior vena cava.

## Supplementary Materials

Supplementary Materials Transesophageal contrast echocardiogram: midesophageal aortic valve short axis view. On the left: superior vena cava injection in the upright position with a small right-left shunt; on the right: inferior vena cava injection in the upright position with a massive right-left shunt.
